# Amplitude modulation structure in French and German poetry: universal acoustic physical structures underpin different poetic rhythm structures

**DOI:** 10.1098/rsos.232005

**Published:** 2024-09-25

**Authors:** Tatsuya Daikoku, Charlotte Lee, Usha Goswami

**Affiliations:** ^1^ Centre for Neuroscience in Education, University of Cambridge, Cambridge, UK; ^2^ Graduate School of Information Science and Technology, The University of Tokyo, Tokyo, Japan; ^3^ Center for Brain, Mind and KANSEI Sciences Research, Hiroshima University, Hiroshima, Japan; ^4^ Faculty of Modern and Medieval Languages and Linguistics, University of Cambridge, Cambridge, UK

**Keywords:** rhythm, speech, phonological hierarchy, amplitude modulation

## Abstract

French and German poetry are classically considered to utilize fundamentally different linguistic structures to create rhythmic regularity. Their metrical rhythm structures are considered poetically to be very different. However, the biophysical and neurophysiological constraints upon the speakers of these poems are highly similar. Scientifically, this suggests that at the level of the acoustic physical structures that are produced orally, the two poetic genres may be rhythmically extremely similar. Here, we apply a language-blind computational model of linguistic rhythm based on features of the amplitude envelope (AE) to compute these physical stimulus characteristics. The model was applied to recordings of the recitation of metrical French and German poems by native speakers. Poems in free verse were not considered in the study. The results indicated that the acoustic physical structures of the poems were identical for the two languages in terms of temporal modulation patterns in the AE. This challenges the linguistic view that German poetry utilizes lexical stress to create prosodic alternation between strong and weak syllables, while French poetry relies on accentuation at the level of prosodic phrasing. Nevertheless, minor differences in physical structure could be detected by applying further modelling drawn, respectively, from the birdsong and neural connectivity literatures.

## 1. Introduction

Poetry, with its inherent artistic qualities, has captivated audiences across cultures and languages for centuries. Poetic metre, or rhythmic linguistic structure, is both pleasing to the listener and more memorable than normal discourse [1]. Yet, it has been long argued that different languages and poetic traditions create rhythmic regularity by different means. In metrical German poetry, as in English, lexical stress (relative within-word syllable prominence) and pitch accents—which shape the stress patterns and phrasal accents—are mobilized to create rhythm patterns. A key structure is the prosodic ‘foot’, which typically has a fixed number of syllables, one of which is more prominent. In metrical French poetry, by contrast, the number of syllables in a line is argued to be the key factor in creating rhythm. Accent (the French equivalent of stress) is more mobile in French than it is in English or German poetry and is thought to occur at the level of phrasing rather than that of individual words.

In this poem by Goethe, an unstressed syllable is followed by a stressed one (marked in bold), giving an iambic rhythm and a foot comprising two syllables:

Ein **Strom** ent**rauscht** um**wölk**tem **Fels**en**saal**e,

Dem **Oz**e**an** sich **ei**lig **zu** ver**bin**den

       (Johann Wolfgang Goethe, ‘Mächtiges Überraschen’).

Similar rhythmic patterns are possible, indeed prominent, in English verse:

If **mu**sic **be** the **food** of **love**, play **on**

       (William Shakespeare, *Twelfth Night*, Act 1, Scene 1, line 1).

French metre, as we have seen, works differently. The traditional alexandrine—a twelve-syllable line which dominated French poetry well into the nineteenth century—has two main accents, on the sixth and 12th syllables, and (usually) two others, which are mobile. Take these lines by Baudelaire:


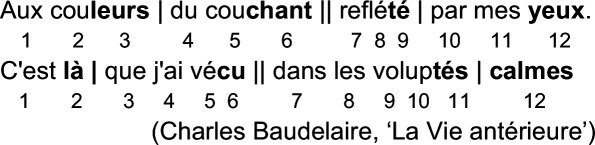


The numbers below each line mark out each syllable. The single ‘bar line’ (∣) , known in French as the ‘coupe’, is simply inserted to help with scansion: it falls after the accented syllables, again marked in bold and indicates the end of one ‘measure’ (mesure). Finally, the double bar (∥) indicates the caesura (pause), which divides the traditional alexandrine into two ‘hemistichs’ (groups of six syllables). This notation makes clear at a glance that there is more variation in the number of syllables between accents (stressed syllables) than there is in the lines from Goethe or Shakespeare discussed above.

There are various reasons why the poetic traditions contrasted here have evolved differently. To an extent, the approaches are consistent with properties of everyday speech. Whereas Germanic languages tend to possess lexical stress, French speakers are considered to approach rhythm in a different way, related to accentuation and phrasing (e.g. [2, p. 238; 3, p. 198]). Phrasal accents can be defined as relative within-phrase word prominence or phrasal stress. Indeed, in experiments, native French speakers seem to exhibit ‘stress deafness’, with no apparent cognitive differentiation of lexical stress in a range of listening tasks (e.g. [4,5, p. 169]). Yet, there may also be other factors in the different trajectories that the poetic traditions have taken: it has been speculated, for example, that taste and aesthetic convention were more significant than intrinsic properties of the language in the development of verse forms and traditions [6, p. 133].

Thus, French poetry has been considered by most critics to be very different from the poetry of the German and English traditions. This is particularly evident where ‘beat’ is concerned. Anglophone poetic and prosodic analysis often foregrounds the notion of ‘beat’ in poetry (e.g. [7]), with the regular alternation of stressed and unstressed syllables enabling listeners to tap along with the alternating stresses just as one may tap to the beat structure in music. German poetry operates in a very similar way. In French, by contrast, ‘beat’ is not considered a factor:

The French accent falls on the last accentuable syllable of each syntactic unit in the line, and since these units naturally vary in length, French rhythmic measures obey no law of recurrence and no principle of regularity, and thus have no connection with the notion of beat. [3, p. 198]

This summary by Scott represents the standard position. Yet, a handful of scholars (above all [8]) have argued that something like the alternating stress patterns characteristic of poetry in German and English can also be discerned in French. Some psycholinguists have also argued that all languages can be described as *more or less stress-timed*, challenging the classical speech rhythm distinction between stress-timed languages (such as German and English) and syllable-timed languages (such as French and Portuguese [9,10]). The logical consequence of all languages being to some extent stress-timed would be that a discernible acoustic ‘beat’ structure would be universally present.

Here, we compare the acoustic ‘beat’ structure of French and German metrical poetry by adopting a language-blind acoustic approach to rhythm patterning. Drawing on neural and acoustic temporal perspectives to beat and rhythm structures, we investigate whether, at the level of the acoustic speech signal that is processed by the brain, the two poetic structures are indeed as different as has been thought. As the brain does not know in advance which language system it will be born into, there may be some acoustic universals regarding human rhythm processing that are reflected in poetry in all languages. While metrical French poetry does not appear on the surface to foster a salient beat, the phrasal accents used in French poetic forms may in fact create physical acoustic structures that, in terms of neural processing, are highly similar to trochaic or iambic forms common in German and English poetry. Following Turner & Pöppel [11], we reason that poetry presents human brains with a temporally, rhythmically and linguistically hierarchical system that is organized to match the hierarchical organizational system of the oscillatory processing networks used by the human brain to encode acoustic sensory information (see [12] for a review). Poetry may thus utilize universal physical structures at the acoustic level, even though the linguistic realization of these physical structures may sound different to the listening human perceiver.

Our experimental approach builds on prior scientific research that suggests that speech and music share similar temporal structures that create the percept of ‘beat’, and that their rhythmic attributes can be described by corresponding linguistic and musical frameworks [13–20]. Our focus here is on the speech amplitude envelope (AE) and the amplitude modulation (AM) patterns nested in the AE, following prior studies of AM patterning in poetry (e.g. [21,22]). It has also been proposed that infants’ early language acquisition begins with the acquisition of universal rhythmic structures, with speech rhythm (known to be perceived by neonates) described as a universal precursor for language acquisition [23,24]. Indeed, the physical acoustic structure (temporal modulation structure) of infant-directed speech (IDS), commonly known as Babytalk, has been shown to match the ‘beat’ structure of many genres of music when analysed from the perspective of the speech AE [20,25]. Converging findings regarding IDS, based primarily on pitch metrics, come from a study comparing speech and song directed to infants versus adults in 21 highly diverse languages [26]. Hilton *et al*. [26] demonstrated that Babytalk across all these languages was characterized by greater pulse clarity and more consistent temporal regularities than speech directed at adults. Clearly, frequency modulation also plays a role in shaping linguistic rhythm perception. Nevertheless, the current modelling builds on our prior work with IDS, child-directed speech (CDS) and music, representing a ‘first pass’ at investigating poetic metre from an AM hierarchy perspective.

The speech AE is the slow-varying energy profile of the acoustic waveform that the ear receives moment-by-moment (amplitude (signal intensity) variation over time [27]), and is crucial for speech intelligibility. A complex signal such as speech or music has many frequencies and the amount of energy in each frequency or frequency band is continually changing over time. The question of whether there is a systematic acoustic structure in the AE of different kinds of speech can be addressed by computational modelling, and such computational modelling of IDS, English nursery rhymes and CDS in Spanish has revealed a universal temporal hierarchical structure of AM that also characterizes many genres of music (e.g. classical, Jazz, song; see [20,25,28,29]). If we assume that poetry can be considered a musical form of language, akin to IDS, then the application of an AE-based computational modelling approach may clarify whether poetry that is classically considered to comprise quite distinct rhythm structures, such as German structures versus French structures, are in fact physically similar.

Our acoustic modelling approach is based on the AM hierarchy of sound waveforms below approximately 40 Hz that comprise the majority of the speech signal analysed by human listeners. AM refers to alternations in sound intensity or loudness in the speech signal stemming from simultaneous movements of the vocal folds, tongue, vocal track, mouth and lips [30]. All these movements together contribute a signal with a varying AE that underpins the perception of speech rhythm and ‘beat’ [30]. Motorically, these movements show high temporal similarity across languages [31]. The core AM bands nested in the speech AE are ‘delta-rate’ modulations (<4 Hz) which support prosodic parsing, ‘theta-rate’ modulations (4–12 Hz), which support syllabic parsing, and ‘beta/low gamma rate’ modulations (12–40 Hz), which help to identify onset phonemes in syllables in English [28]. The labels delta, theta, beta and gamma are drawn from neurophysiology, as cell networks that oscillate at these temporal rates are found across the brain. In the auditory cortex, these oscillatory rates are important for speech processing [12,32]. Notably, regarding the physical structure of rhythmic acoustic signals, a matching AM hierarchy is detectable in music as well as in other languages including Portuguese and Spanish [20,29,33]. No linguistic study has yet examined the hierarchical AM structure of other European languages such as German and French. We contribute such a study here.

In the present study, we analyse the hierarchical temporal structure of read-aloud poetry in French and German. These two languages are spoken in geographical proximity and have considerable overlap in terms of literary history, but are considered to have fundamentally different rhythmic structures at all levels, from everyday speech to metrical poetry. Our primary interest is in whether modelling the acoustic structure of metrical poetry in each language will reveal nested hierarchical temporal modulation structures in the AE that are more similar than different. To analyse temporal modulation structure, we applied the spectral-amplitude modulation phase hierarchy (S-AMPH) model [28,34]. The S-AMPH model analyses the AM structure of the AE of any sound by separating the AM characteristics from the frequency modulation (FM) characteristics. S-AMPH generates a low-dimensional representation of the speech signal consisting of the dominant spectral (acoustic frequency, including pitch and formant) and temporal (oscillatory rate and speech rhythm) modulation patterns in the speech envelope. This approach enables us to observe which *timescales* of rhythmic modulation are predominant in each type of poetic genre. The S-AMPH model was first applied to English nursery rhymes and subsequently to IDS (Babytalk, see [25]). Previous research using the S-AMPH modelling approach has demonstrated similar AM bandings in different languages, with three core AM bands centred, respectively, on approximately 2, 5 and 20 Hz, along with more modulation energy in the approximately 2 Hz AM band found in IDS. The greater modulation energy in the delta-rate AM band implies more exaggerated prosodic stress in spoken IDS compared to adult-directed speech (ADS). Prior modelling studies also revealed more rhythmically structured input in music and IDS than in natural ADS (indicated by significantly greater phase relations between the delta and theta AM bands; see [20,25,29]). The phase relations between both the delta- and theta-rate AM bands and the theta- and beta-low gamma-rate AM bands also become greater in ADS the more literate the participants [33].

Accordingly, in light of this prior literature with European languages, we predict that the number of bands of AM and their boundaries (i.e. the temporal modulation structure of the poems) will be very similar for spoken French and German to the AM bands identified previously for conversational English (IDS, ADS) and Portuguese (ADS only), and for rhythmic CDS in English and Spanish. We also predict a similar set of AM phase relations for both German and French, with the delta- and theta-rate AM bands exhibiting the largest phase synchronization index (PSI). Significantly greater delta–theta AM phase alignment was found previously for both different types of rhythmic speech (English nursery rhymes, English IDS and Portuguese proverbs) and for music. Regarding the neural encoding of these AM bands, the neural oscillators are known to be dynamically modulated from slower to faster bands in a top-down manner [32,35]: delta oscillators modulate the theta oscillators, and theta oscillators modulate the gamma oscillators. Such a ‘cascade’ oscillatory system is reflected in the phonological AM hierarchy generated by the S-AMPH, in which delta-rate AMs also sit at the apex. As the brains and the speech comprehension and production mechanisms of the listeners of the poems are biophysically highly similar, this neural consideration further motivates our *a priori* hypothesis that the physical stimulus characteristics of the AE which define rhythm patterns in French versus German metrical poetry will be more similar than different. In particular, we predicted similar AM structures in terms of the constituent AM bands and their boundaries, and the largest PSI for the delta-rate and theta-rate AM bands. In particular, a PSI of 1:2 would indicate the presence of a clear ‘beat’.

However, it is also the case that most listeners can clearly perceive the difference between German poems, with their alternating stresses and French poetry. To try and locate the physical source of these perceived differences, we also applied a type of modelling used to compare the physical characteristics of rhythm patterns in human music versus birdsong [31,36]. This alternative modelling approach contrasts ‘horizontal rate’ patterns such as 1:1 (metronome-like) beat structures versus 1:3 (triplet) beat structures, and ‘vertical ratios’, which identify the relations between cycles of rhythmic patterns at different hierarchical levels of a piece of music or the song of a bird. Our expectation was that given that audiences can distinguish between French and German poetry, the recited speech might differ in terms of either the horizontal rate patterns, or the vertical ratio patterns, or both. Finally, following the neuroscience literature on transfer entropy, we attempted to predict whether the metrical structure of one cycle of the prosody waveform can help to predict the metrical structure of the syllabic waveform that lies within the subsequent cycle of the prosodic waveform. For example, if a top-down transfer entropy from prosodic to syllabic bands exceeds a bottom-up transfer entropy from syllabic to prosodic bands, this would constitute a compelling argument that the prosodic rhythm is a good predictor of the syllabic rhythm. Given the use of syllable counting in French poetry and the use of lexical stress in German to create rhythm, we tentatively predicted that we would find higher top-down transfer entropy in German metrical poetry than in French metrical poetry.

## 2. Material and methods

### 2.1. Participants

A total of 22 adults were recruited for this study, consisting of 10 native speakers of German (six women and four men) and 12 native speakers of French (nine women and three men). All participants were raised in a monolingual context until they were introduced to foreign languages at school, typically around the age of 10 years, and were associated with the University of Cambridge as adults. Ethical review for this study was conducted by the Cambridge Psychology Research Ethics Committee. Informed consent was obtained from all participants. The participants had a range of exposure to poetry. All had learned and analysed some at school, but some had not encountered it since and had no professional involvement with either literature or language, while others had studied or were studying the literature of their native language at undergraduate or even postgraduate level.

### 2.2. Selection of poems

Twenty-five poems were selected in each language by C.L., a specialist in poetic traditions: ten nineteenth-century sonnets, five baroque sonnets and ten further poems in a variety of forms for each language. The syllable counts and text excerpts of each poem can be found in the electronic supplementary material (appendix S1). The poems, as presented in the electronic supplementary material, appendix S1, and as explained in further detail below, have been matched carefully across the languages: it was thus deemed crucial for each speaker to read the poems in the same order. No attempt was made to match the thematic content of the poems: the experimental considerations were purely structural.

The poems chosen are all highly structured. Nineteenth-century poems predominate in the corpus because, in this period, poets in both German and French were still committed to regular metres, while at the same time, demonstrating flexibility and a willingness to experiment with, even to challenge, fixed forms. The nineteenth-century sonnets are in iambic pentameter in German and in alexandrines in French. The baroque sonnets were included because some early modern German-speaking poets adopted the 12-syllable alexandrine form before French influences were displaced by others (above all English). The sonnets by Gryphius and Ronsard, therefore, share many of the same structural features: 12 syllables, medial caesura. Even when adopting this form, however, German poets such as Gryphius tended to follow the preference of the poet and theorist Martin Opitz for an accentual-syllabic line. Overall, the corpus offers a balance of constants (five poems in alexandrines in both languages, 15 sonnets in both languages) and contrasts (French alexandrines versus German iambic pentameters in the nineteenth-century sonnet group, the variety of the final 10 poems both within and across the language groups).

A key consideration was finding pairs of French and German poems which were as close in overall duration (i.e. time taken to read the poem) as possible. This was achieved by matching both the number of lines and the approximate syllable count of the poems across languages. Syllable count could not be perfectly matched due to the constraints inherent in accented phrasing. Clearly, the duration of a poem will also be affected by the lengths of individual syllables, and by the idiosyncratic speeds adopted by different speakers of the poems. Nonetheless, approximately matching the syllables was the most systematic approach that could be taken, and it is an appropriate approach given versification systems in both French (where the syllable count is paramount) and German (where stress predominates but syllable count is still significant in many metres). Most of the poems also tend to have lines that are end-stopped: that is, where there is a slight pause at the end of each line before the metre resumes. These regular pauses help to create an equivalent (though, as ever, not equal) sense of rhythmic shape and expectation between poems. Poems with enjambement have been matched to one another across languages, although the enjambement cannot always be at the same point in the respective poems.

The German poems are all in duple rhythms—that is, they are formed predominantly of bi-syllable ‘feet’, where one unstressed syllable alternates with one stressed syllable. (An extra syllable may creep in occasionally for effect, but this does not alter the nature of the rhythmic base.) The majority of the poems are iambic (unstressed–stressed), with four examples (‘Die Nachtblume’, ‘Herr! ich steh in deinem Frieden’, ‘April’, ‘Zwielicht’) of trochaic metre (stressed–unstressed). Triple rhythms, which are formed of tri-syllable feet such as dactyls (stressed–unstressed–unstressed), anapaests (unstressed–unstressed–stressed) and amphibrachs (unstressed–stressed–unstressed), have not been included, nor have mixed forms such as the dolnik, which shift between duple and triple (an example would be Goethe’s ‘Erlkönig’, not represented in this corpus). The French poems will inevitably display more rhythmic variation: as is evident from the examples discussed at the start of this article, the number of syllables in a syntactic unit (‘measure’, or ‘mesure’ in French) is variable, and the notion of a prosodic ‘foot’ with a fixed number of syllables does not apply.

Considerable care was taken to create a corpus that could offer genuine points of comparison (sonnets, alexandrines, line length and syllable count) without imposing an artificial sense of uniformity. Poetic rhythm is, by its nature, highly variable. We have just seen, in our discussion of measures and feet, that the ‘building blocks’ of metre are different in German and in French. Moreover, even poetry which does conform to a recognizable metrical pattern will likely exhibit internal tensions and variations, due in part to grammatical considerations. Take the lines from Goethe quoted at the start of this paper, ‘Ein Strom umrauscht umwölktem Felsensaale/Dem Ozean sich eilig zu verbinden’: these are iambic lines (unstressed–stressed), yet the words ‘Felsen’ and ‘-saale’ are naturally trochaic (stressed–unstressed), creating a kind of counterpoint with the iambic base. This sense of counterpoint occurs frequently in German poetry [37,38]. Moreover, this line ends on an unstressed syllable (known as a feminine ending), while others in the corpus end on stressed syllables (masculine endings). Finally, the degree of stress will not be exactly the same for each syllable: in ‘Ozean’, for example, the stress on ‘O’ will likely be greater than that on ‘an’, while the stress on ‘zu’ will be less prominent than on ‘bind’ (in ‘ver**bind**en’). Such variability is inherent in language, but it is also part of the art form. It would be impossible to control fully the differences between poems and across languages, nor would it be desirable. The purpose of this study is to make salient the natural continuities in acoustic structures that emerge in material which—in equally natural fashion—is heterogeneous.

### 2.3. Procedure

Each participant was provided in advance with a printed copy of the 25 poems in their native language. Upon arrival at the laboratory, they were instructed to read the poems aloud inside a soundproof room, standing up and speaking into a microphone. Their speech was recorded. Participants had control over the recorder; pressing pause after each recording created a natural break, which they were advised to use to familiarize themselves with the next poem, to rest, to drink, etc. They were encouraged to read naturally rather than to ‘perform’ the poems, though their attention was drawn to stylized elements, above all the need to pronounce the French *e muet*, or silent ‘e’, in certain positions in the poetic line, which may differ from a speaker’s instinct in everyday spoken French. This particular intervention was considered necessary because, without it, syllable count (a key factor in creating some degree of continuity across the poems) would have been affected. Participants were asked to start reading a poem again from the start if they stumbled; interrupted versions were then deleted from the data.

The speech data were then analysed to examine the AM structures in the AE of the speech waveforms for both the German and French native speakers. A detailed summary of the reading time data, including mean values and standard deviations, can be found in table 1.

**Table 1.** Mean sample duration for recitation of poems in French and German.

	French	German	*t*(20)	*p*	95% CI		Cohen’s *d*
					lower	upper	
*N* (females)	12 (9)	10 (6)	—	—	—	—	—
mean duration (s)	47.4 (±7.51)	50.7 (±7.11)	−1.02	0.33	−10.3	3.61	−0.44
Shapiro–Wilk *W*	0.92	0.94	—	—	—	—	—
Shapiro–Wilk *p*	0.33	0.60	—	—	—	—	—

Averages are reported as mean ± s.d.

### 2.4. Data analysis

**Figure 1.**
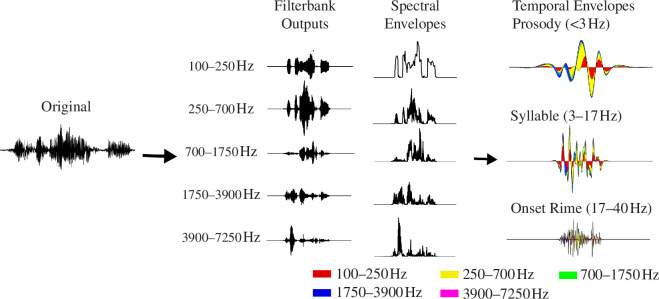
Signal processing steps in S-AMPH model. The original waveform is an example of the poetry samples used in this study. It is passed through an ERB_N_-spaced filter bank (step 1), yielding a hierarchical representation of the core spectral (acoustic frequency spanning 100−7250 Hz) and temporal (oscillatory rate spanning 0.9−40 Hz) modulation hierarchies in the AEs of speech (labelled spectral envelopes and temporal envelopes, respectively). The envelope is extracted from each channel output using the Hilbert transform. The number and the edges of bands are determined by PCA dimensionality reduction of original spectral and temporal envelope representations to identify patterns of covariation across spectral and temporal channels. Finally, the S-AMPH model can generate a cascade of amplitude modulators at different oscillatory rates, producing the AM hierarchy. For our example poem, the figure shows five core spectral bands (band 1: 100−250 Hz, band 2: 250−700 Hz, band 3: 700−1750 Hz, band 4: 1750−3900 Hz, band 5: 3900−7250 Hz) and three temporal bands (prosodic rate: 0.9−3, syllabic rate: 3−17 and phonetic rate: 17−40 Hz). Spectral band 1 covers the typical pitch frequency of the human voice [39], while spectral bands 2 and 3 are typical second and third formant frequencies of the human voice [40]. We also analysed the mean frequency power (MFP) to understand which spectral band most strongly contributes to temporal amplitude structure. For more details, see electronic supplementary material, appendix S2.

Detailed modelling methodologies are reported in [28] (for wiki, see https://www.cne.psychol.cam.ac.uk). This approach extracts a hierarchy of dominant AM patterns in different speech styles. This is conducted by first creating high-dimensional representations of the acoustic signal and then applying principal component analysis (PCA) to the spectral and temporal representations (herein, spectral and temporal PCA, respectively). Thus, yielding the core modulation bands in each speech register (figure 1).

#### 2.4.1. Spectral amplitude modulation phase hierarchy

The acoustic speech signals were normalized based on z-scores (*M* = 0, s.d. = 1) in case the sound intensity influenced the spectrotemporal modulation feature. The raw acoustic signal was passed through a 28 log-spaced ERB_N_ filter bank spanning 100−7250 Hz, which simulates the frequency decomposition by the cochlea in a normal human to establish the patterns of spectral modulation [41,42]. For further technical details of filter bank design, see Stone & Moore [43]. The parameters of the ERB_N_ filter banks and the frequency response characteristics are provided in electronic supplementary material, appendix S2. The envelope for each of the 28 filtered signals was obtained by the 28 Hilbert transform. Using the 28 Hilbert envelopes, core spectral patterning was defined by PCA. This can identify the appropriate number and spacing of non-redundant spectral bands by detecting co-modulation in a high-dimensional ERB_N_ representation. The raw acoustic signal was filtered into several spectral bands identified in the spectral PCA to establish temporal modulation patterns.

In the spectral PCA, only the top five principal components (PCs) were considered for further analysis (figure 1, middle) because they already cumulatively accounted for over 65% (on average) of the total variance in the original sound signal. Matching spectral bands have been found in S-AMPH studies of other languages [29,33], and are likely linked to features of the human voice. Then, the AM envelope in each spectral band was passed through a 24 log-spaced ERB_N_ filter bank spanning 0.9−40 Hz (electronic supplementary material, appendix S2). The envelopes for each of the 24 filtered signals were obtained using Hilbert transform. Core temporal patterning was also defined using temporal PCA. We also analysed the mean frequency power (MFP) to determine which spectral band strongly contributes to the temporal amplitude structure.

In the temporal PCA, only the top three PCs were considered for further analysis because they cumulatively accounted for over 90% of the total variance in the original sound signal (figure 1, right). Details of the PCA results are provided in electronic supplementary material, appendix S3. The absolute values of the PC loadings were averaged across all the speech samples. Then, the peaks in the grand average PC loading patterns were obtained to identify the core modulation hierarchy. Troughs were also identified because they reflect the boundaries of the edges between co-modulated clusters of channels [20].

PCA has previously been used for dimensionality reduction in speech studies (e.g. [44,45]) This study focused on the absolute value of component loadings rather than component scores. The loadings indicate the underlying patterns of correlations between high-dimensional channels. That is, PCA loading was adopted to identify patterns of covariation between the high-dimensional channels of spectral (28 channels) and temporal (24 channels) modulations and to determine groups (or clusters) of channels that belonged to the same core modulation bands (see electronic supplementary material, appendix S2, for more details).

We set criteria to determine the core spectral and temporal modulation bands based on previous studies using S-AMPH [20,28]. For instance, to ensure adequate spacing between the resulting inferred modulation bands, a minimum peak-to-peak distance of two and five channels was set for the spectral and temporal PCAs, respectively. Furthermore, the cycles in which the peak-to-peak amplitude was less than 10% of the mean peak-to-peak amplitude (i.e. the difference between peak and trough) were excluded because they mostly represent noise but not speech cycles. After detecting all the peaks and troughs, the core spectral and temporal modulation bands were determined based on the criteria that at least two of the five PCs and one of the three PCs showed a peak for the spectral and temporal bands, respectively. On the other hand, the boundary edges between modulation bands were determined based on the most consistent locations of ‘*flanking*’ troughs for each group of PC peaks that indicated the presence of a band.

#### 2.4.2. Phase synchronization analyses

We investigated the multi-timescale phase synchronization between bands by computing the ratios between ‘*adjacent*’ bands. These ratios were computed for each pairing of the temporal modulation bands generated by the application of the S-AMPH model to the poems in each language. The PSI was computed between adjacent AM bands in the S-AMPH representation (i.e. delta versus theta, theta versus beta/gamma phase synchronizations). The *n*:*m* PSI was initially conceptualized to quantify phase synchronization between two oscillators of different frequencies (e.g. muscle activity [46]) and was subsequently adapted for neural analyses of oscillatory phase-locking [47]. PSI was computed as follows:


(2.1) $$PSI=|e^{1(n\theta 1-m\theta 2)}|$$

where *n* and *m* represent the relative difference in the cycle length describing the frequency relationship between the lower and higher AM bands. For example, if the cycle length in the temporal information of delta rhythm is 2000 ms and the cycle length of theta rhythm within the delta rhythm is 1500 ms, the *n*:*m* ratio for PSI is 4:3. The *n*:*m* ratio was calculated for each PSI.

The values *θ*1 and *θ*2 refer to the instantaneous phases of the two AMs at each time point. Therefore, (*nθ*1 − *mθ*2) is the generalized phase difference between the two AMs, computed using the circular distance (modulus 2π) between the two instantaneous phase angles. The angled brackets denote the average of this phase difference over all the time points. PSI is the absolute value of this average and can take values between 0 and 1 (i.e. no to perfect synchronization [25]). A sound with a PSI of 1 is perceived as perfectly rhythmically regular (a repeating pattern of strong and weak beats). Simultaneously, a sound with a PSI of 0 is perceived as random in rhythm.

#### 2.4.3. Horizontal rate patterns and vertical ratios

We investigated the horizontal rate patterns at each AM hierarchy level and the vertical ratios between levels by computing the cycle lengths of the AM waveform corresponding to the prosodic (delta) and syllabic (theta) levels. This analysis also adopted the 3 × 5 temporal modulation envelopes in the S-AMPH model. After isolating the AM bands, the cycle lengths were assessed.

First, troughs were identified because they reflected the boundaries of the edges between cycles. After detecting all troughs in each of the temporal rate bands (delta and theta/alpha), the cycle lengths were determined by calculating the length between adjacent troughs. Then, we calculated the horizontal rate and vertical ratios. These analyses were restricted to the AM delta and theta bands. For example, if the length of a prosodic cycle (delta AM p_1_) is 500 ms and the length of the subsequent prosodic cycle (delta AM p_2_) is 500 ms, then the horizontal rate is 0.5 (i.e. 1:1) based on the formula of p_1_/(p_1_ + p_2_) [31]. Furthermore, if the length of a prosodic cycle (delta AM p_1_) is 500 ms and the length of the syllabic cycles (e.g. theta AM s_1_, s_2_) within the time window of this delta cycle (p_1_) is 250 ms, the vertical ratio is 0.5 (i.e. 1:2) based on the formula of s_1_/p_1_, s_2_/p_1_. These values were calculated for each prosodic cycle in each poem.

We then averaged the cycle lengths per poem and averaged across the five spectral bands in each language. Then, we performed the Shapiro–Wilk test for normality on the difference from the 1:1 horizontal rate (absolute value of 0.5 ×) and the difference from the 1:2 vertical ratio (absolute value of 0.5 ×). Depending on the result of the test for normality, either the parametric or non-parametric (Kruskal–Wallis) one-way analysis of variance (ANOVA) including a between-group factor (German and French) was applied. Statistical analyses were conducted using jamovi Version 1.2 (the jamovi project, 2021). We selected *p* < 0.05 as the threshold for statistical significance.

#### 2.4.4. Transfer entropy analyses

Finally, we investigated the information dynamics among the delta, theta and beta/low gamma AM bands, namely the prosodic, syllabic and phonetic rhythms. We applied a transfer entropy analysis to understand these dynamics. Transfer entropy is a non-parametric statistic that can measure the dynamic properties of two variables [48]. Transfer entropy can measure the directionality of information dynamics from a specific phonological cycle, such as prosody, syllable and phoneme, to another cycle. For example, a high value of transfer entropy from prosodic to syllabic AM envelopes (delta band to theta band cycles) suggests that speech rhythm is transferred from prosodic to syllabic levels. More specifically, transfer entropy from a variable *X* to another variable *Y* is the amount of uncertainty reduced in future values of *Y* by knowing the past values of *X* given the past values of *Y*. The transfer entropy can also be expressed as


(2.2) $$T_{X\to Y}=H(Y_{t}|Y_{t-1:t-L})-H(Y_{t}|Y_{t-1:t-L},X_{t-1:t-L}),$$

where *H*(*X*) is the entropy of *X* based on information theory [49]. Previous neural evidence suggests that the oscillators are hierarchically and dynamically modulated from slower to faster bands in a top-down manner [32,35]. Accordingly, we hypothesized that the dynamical system from slower to faster speech rhythm cycles may play a key role in speech hierarchical structure.

We set the time window for the transfer entropy analysis based on the results obtained from the temporal PCA using S-AMPH and the FFT analysis of prosodic waveforms derived from the 3 × 5 temporal modulation envelopes based on five spectral bands and three temporal modulation bands (see §3). We then averaged the value of transfer entropy across the five spectral bands in each language. Then, we performed the Shapiro–Wilk test for normality on the transfer entropy. Depending on the result of the test for normality, to examine the directionality in each pair of hierarchy (i.e. prosody–syllable, prosody–phoneme and syllable–phoneme), either the parametric or non-parametric (Friedman) repeated measures ANOVA was applied. Furthermore, to examine the group difference in each transfer entropy, either the parametric or non-parametric (Kruskal–Wallis) ANOVA was applied. Statistical analyses were conducted using jamovi Version 1.2 (the jamovi project, 2021). We selected *p* < 0.05 as the threshold for statistical significance and used a Durbin–Conover method for *post hoc* analysis and multiple testing of significant effects.

## 3. Results

### 3.1. Amplitude modulation properties

**Figure 2.**
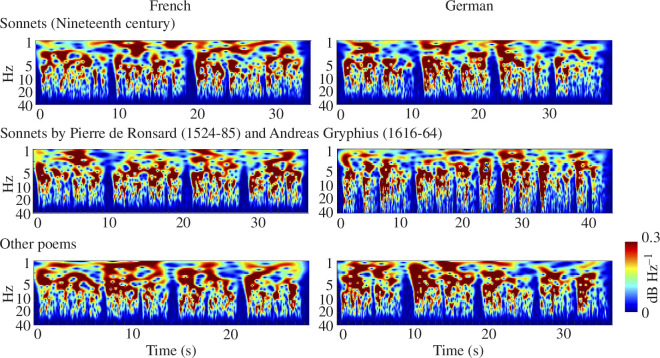
Scalograms depicting the AM envelopes of the poetic material. We depict a representative poem in each category of nineteenth-century sonnets, baroque sonnets and other poems using continuous wavelet transform (CWT), which was run on each AM envelope from randomly chosen 30 s excerpts of speech. Note that similar scalograms cannot be generated for S-AMPH because of the use of cochlear filterbanks, which means that boundary frequencies would disappear. The *x*-axis denotes time (30 s) and the *y*-axis denotes modulation rate (0.1–40 Hz). The maximal amplitude is normalized to 0 dB. The demodulation outputs are shown as a heat map.

To provide a visualization of the overall acoustic structures of the poems modelled here, we provide scalograms showing the averaged data as figure 2. Wavelet heatmaps act as an easily interpretable visual method, capturing the temporal dynamics essential to poetic expression. The figure suggests high acoustic similarity between French and German, which is explored statistically below.

#### 3.1.1. Spectral principal component analysis

The first to fifth PCs (PC1–PC5) accounted for an average of 29.40%, 16.00%, 7.69%, 6.01% and 5.09% of the total variance in French, respectively. PC1–PC5 accounted for an average of 31.11%, 16.20%, 8.35%, 5.88% and 4.78% of the total variance in German, respectively. Each group’s grand average, loading patterns and cumulative contribution were deposited to an external source (https://osf.io/9mtke/?view_only=3cfc2c3d30824674836f4216fab15159). One peak (approximately 3000 Hz) was identified in the loading pattern of PC1 (electronic supplementary material, appendix S3). The loading pattern is virtually identical to the mean correlation coefficient between each and all other spectral channels. Thus, PC1 was assumed to reflect the global correlation between the spectral channels. The peak of approximately 200 Hz and the ‘*flanking*’ trough of approximately 250–300 Hz were identical between PC2 and PC3. This provides corroborating evidence for the lowest spectral band at this spectral location with a potential boundary between the first and second spectral bands at approximately 250 Hz (troughs indicate potential boundaries between modulation rate bands). Further peaks and troughs were identified, providing evidence for four additional spectral bands (see electronic supplementary material, appendix S3 and Methods). Based on the *a priori* criteria (see §2), the spectral PCA provided evidence for the presence of five core spectral bands in the spectral modulation data (band 1: 100−250 Hz, band 2: 250−700 Hz, band 3: 700−1750 Hz, band 4: 1750−3900 Hz, band 5: 3900−7250 Hz). At least two of five PCs showed peaks in these five spectral regions. Furthermore, we consistently observed four boundaries between the five spectral bands (250, 700, 1750 and 3900 Hz). Table 2 summarizes the five spectral bands and their boundaries for each language. Notably, these five spectral bands are proportionately scaled concerning the logarithmic frequency sensitivity of human hearing. As predicted, these results were similar to the spectral bands previously revealed by modelling IDS in English, CDS in English and Spanish, ADS in English and Portuguese and music [20,25,28,29,33].

**Table 2.** Summary of the five spectral bands and the four flanking boundaries indentified from spectral PCA for each language.

language	spectral bands	frequency range (Hz)	PC peaks
German	band 1	100–250	PC1–PC3
	band 2	250–700	PC2–PC5
	band 3	700–1750	PC2–PC3
	band 4	1750–3900	PC1, PC3–PC5
	band 5	3900–7250	PC2–PC5
French	band 1	100–250	PC2–PC3
	band 2	250–700	PC3–PC4
	band 3	700–1750	PC2–PC3
	band 4	1750–3900	PC3–PC4
	band 5	3900–7250	PC2–PC5

#### 3.1.2. Temporal principal component analysis

**Figure 3.**
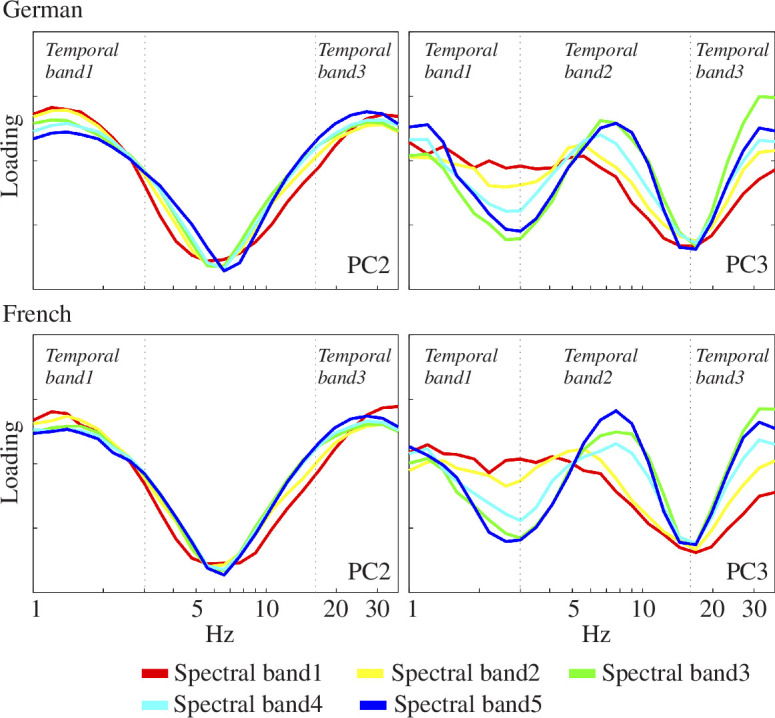
Core temporal modulation rates by language. The average absolute value of the temporal PCA component loading patterns for PC2 and PC3 generated by the S-AMPH model is depicted. The model showed an AM hierarchy that consisted of delta-, theta- and beta/gamma-rate AM bands. Colours in the figure represent the five spectral bands (spectral band 1: 100−250 Hz, spectral band 2: 250−700 Hz, spectral band 3: 700−1750 Hz, spectral band 4: 1750–3900 Hz, spectral band 5: 3900−7250 Hz). The core temporal modulation rates are similar between German and English, and distributed across three temporal bands (i.e. temporal band 1: 0.9–3 Hz, temporal band 2: 3–17 Hz, temporal band 3: 17–40 Hz).

Figure 3 shows the grand average loading patterns (absolute values) for the first three PCs arising from the temporal PCA of each of the five spectral bands determined in the spectral PCA (electronic supplementary material, table a in appendix S3). As can be observed, the loading exhibited consistent patterns among the three PCA loading patterns.

The first to third PCs (PC1–PC3) accounted for 63.99%, 8.36% and 3.78% in French, respectively. PC1–PC3 accounted for 63.80%, 7.97% and 3.88% in German, respectively. Each language group’s grand average, loading patterns and cumulative contribution were deposited to an external source (https://osf.io/9mtke/?view_only=3cfc2c3d30824674836f4216fab15159). PC1 exhibited a moderate peak at 7 Hz acoustic frequencies in all five spectral bands (electronic supplementary material, figure b in appendix S3). As observed in the spectral PCA, PC1 in the temporal PCA may reflect the global correlation between temporal channels. As no troughs were detected in PC1 (indicating no potential boundaries), our analysis focused on PC2 and PC3 (figure 3). The loading patterns of PC2 resulted in two strong peaks at acoustic frequencies of 1.5 Hz (evidence for a delta-rate band of AMs) and 25 Hz (evidence for a beta–gamma rate band of AMs) and one strong flanking trough at acoustic frequencies of approximately 7 Hz. These findings were consistent among the five spectral bands, suggesting the potential existence of at least two core temporal bands. Compared with PC1 and PC2, PC3 loading patterns varied across spectral bands. The spectral bands other than band 1 showed a peak in loading at approximately 1.5 Hz and 7–8 Hz, and all spectral bands showed a peak at approximately 30 Hz. The flanking troughs for these peaks occurred at 3 and 17 Hz. Based on the *a priori* criteria (§2), temporal PCA provided evidence for the presence of three core bands with two boundaries (see table 3). These AM bands also matched prior studies on ADS, CDS, IDS and music [20,25,28,29,33].

**Table 3.** Summary of the three temporal bands and the three flanking boundaries identified from temporal PCA for each language.

language	temporal bands	frequency range (Hz)	PC peaks
German	band 1	0.9–3	PC2, PC3 in spectral bands 1–5
	band 2	3–17	PC3 in spectral bands 2–5
	band 3	17–40	PC2, PC3 in spectral bands 1–5
French	band 1	0.9–3	PC2, PC3 in spectral bands 1–5
	band 2	3–17	PC3 in spectral bands 2–5
	band 3	17–40	PC2, PC3 in spectral bands 1–5

### 3.2. Multi-time scale phase synchronization

To explore the degree of phase synchronization between bands, we generated the 3 × 5 temporal modulation envelopes based on five spectral bands and three temporal modulation bands for each language. It should be noted that the MFP of spectral band 1 (pitch band in human voice) and band 2 (pitch-formant band) is much higher than in the other bands (electronic supplementary material, appendix S4), suggesting that these bands strongly contribute to speech rhythm.

Next, we investigated multi-timescale phase synchronization (PSI) [25] between *adjacent* AM bands in the hierarchy. We also examined the integer ratios between adjacent AM bands to establish the strength of ‘beat’. Given previous evidence that rhythmic speech in English and Portuguese (classically considered to be stress-timed and syllable-timed, respectively) exhibits significantly stronger phase synchronization between the slower delta- and theta-rate AM bands than between the theta- and beta-low gamma rate AM bands, we had hypothesized that the phase synchronization between the delta and theta AM bands (approximately the prosodic and syllabic rhythms) should be the strongest in both German and French. Furthermore, given prior analyses with different musical genres we had expected to find that the integer ratio of 1:2 should be the strongest when a metrical beat structure is present.

The PSI analyses revealed high consistency between participants for the PSIs (see electronic supplementary material, appendix S4). Therefore, further analysis focused on the grand average. The results showed that the PSI of 1:2 integer ratios was, as predicted, the highest PSI in the delta-theta AM bands for German poetry, and indeed for French. The PSIs of 1:3 integer ratios for the S-AMPH modelling were also higher than the other integer ratios, suggesting that for poetry the simpler integer ratios (i.e. *m*/*n*) were most likely to synchronize between adjacent bands. These findings are consistent with our prior findings for IDS, rhythmic CDS and music [20,25,28,29].

### 3.3. Horizontal rate and vertical ratios

Although the core temporal modulation structures of French and German poetry are thus highly similar, it is still the case that listeners can distinguish between them. Accordingly, we also investigated relations between the different acoustic cycles identified by the S-AMPH model in terms of quantifying horizontal relations within a rhythmic cycle (e.g. within the prosodic rate) and vertical relations between cycles (e.g. between the prosodic and syllabic rate). All of the results of statistical analyses and the descriptives of the horizontal rate and vertical ratios have been deposited to an external source (https://osf.io/9mtke/?view_only=3cfc2c3d30824674836f4216fab15159). The averaged data by language are shown in figure 5. The plots indicate that the probability densities of a 1:1 horizontal rate and 1:2 vertical ratio were statistically stronger than the other rates and ratios (see shading in Figure 5). Accordingly, both types of poetry show a clearly discernable metronome-like beat rate (1:1 horizontal rate), and share similar numbers of syllables in a prosodic cycle (typically 2 syllables per cycle; 1:2 vertical ratio).

These rates and ratios were then compared across languages. As the Shapiro–Wilk test detected non-normal distributions for each dataset regarding both rates and ratios (*p *< 0.05), we utilized non-parametric ANOVAs. The DV in each case was a single number representing ratios or rates and the between-group factor was language (German versus French). Vertical ratio indicated that the probability densities of the 1:2 vertical ratio were significantly stronger in German than in French poetry (*χ*² *=* 2.62, *p =* 0.048, *ε*² *=* 0.007). This indicates that the length of a syllabic cycle is more likely to be half of a prosodic cycle in German than in French, although the effect size was small. This indicates that overall the prosodic cycles in French and German poetry are equivalent.

### 3.4. Transfer entropy analysis

Finally, we applied an analysis technique drawn from systems neuroscience to explore whether the directionality of information dynamics from a specific acoustic AM cycle, such as a prosodic cycle, could predict the structure of another AM cycle, such as a syllabic cycle. A transfer entropy analysis was applied to all three cycles in the AM hierarchy identified here, prosodic, syllabic and phonemic, to explore their dynamic inter-relations.

**Figure 4.**
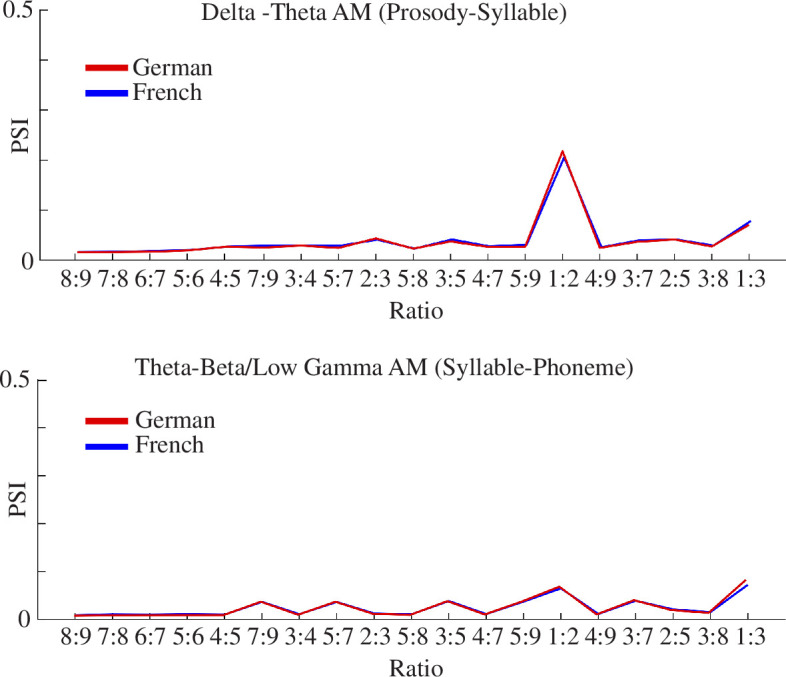
Phase synchronization is averaged across the five spectral bands for each language. The 1:2 ratios are as prevalent in the French poems as in the German poems in this corpus.

**Figure 5.**
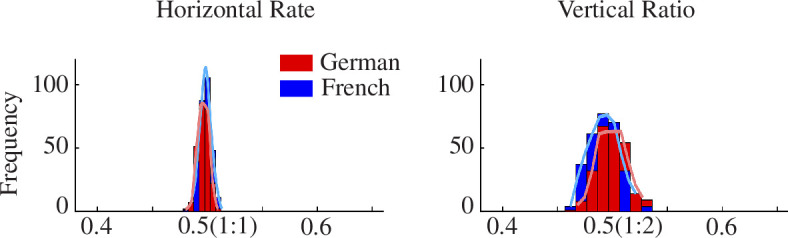
Histogram for the prosodic rate and prosodic–syllabic ratios in each Language. The *y*-axes show the prosodic rates (left) and the prosodic–syllabic ratios (right). The *x*-axes show the frequency (i.e. number) of each prosodic rate and prosodic–syllabic ratio. Based on the formula of p_1_/(p_1_ + p_2_), the 0.5 (1:1) horizontal rate indicates that the relationship between the length of a given prosodic cycle (delta AM p_1_) and that of the subsequent prosodic cycle (delta AM p_2_) is equivalent, while a 1:2 rate refers to a situation where the length of the succeeding prosodic cycle is twice that of the preceding prosodic cycle. Furthermore, based on the formula of s_1_/p_1_, the 0.5 (1:2) vertical ratio refers to a situation where the length of the syllabic cycle is half that of the prosodic cycle. The 1:1 horizontal rate and 1:2 vertical ratio were statistically stronger than the other rates and ratios.

Based on the results obtained from the PSI analysis (figure 4) and the findings of the vertical ratio analyses (refer to figure 5), it is evident that there are typically two syllables within a single prosodic cycle in each language (i.e. 1:2 prosodic–syllabic ratio), while the horizontal rate analyses suggest a 1:1 prosodic rhythm like an alternating beat, again present in both languages. This suggests that one cycle of the prosody waveform can help to predict the syllabic waveform that lies within the subsequent cycle of the prosodic waveform in both languages. We had expected *a priori* that prosody will exert top-down control over syllable placement in both languages. We hypothesize that the prediction of syllabic rhythm might be facilitated by incorporating information from the higher level of prosodic rhythm in a top-down manner in both French and German, which would be evidenced by a stronger T statistic (T (prosody→syllable)) in transfer entropy analyses.

Based on the results obtained from the FFT analysis of prosodic waveforms (electronic supplementary material, figure a in appendix S4), the average frequency of the syllabic rhythm was approximately 4 Hz (250 ms). This suggests that two syllabic cycles in one prosodic cycle correspond to approximately 500 ms. Therefore, we set 500 ms (two-syllabic cycle length) as the time window for the transfer entropy analysis and computation of the T statistics.

**Figure 6.**
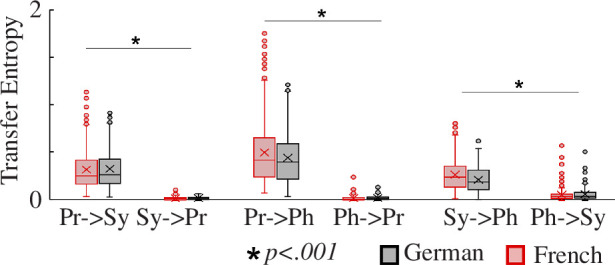
Information dynamics from one level in the temporal hierarchy to another level. Pr = prosody, Sy = syllable, Ph = phoneme. Pr->Sy indicates transfer entropy T (prosody → syllable): information dynamics from prosody to syllable. For example, a high value of transfer entropy from prosodic to syllabic rhythm can be interpreted as showing that the direction of the information dynamics is from prosodic rhythm cycles to syllabic rhythm cycles. The prosodic structure of a given cycle predicts the arrangement of syllables in the following prosodic cycle.

The Shapiro–Wilk test detected non-normal distributions regarding each dataset (*p* < 0.05). Therefore, we utilized non-parametric Friedman repeated measures ANOVAs for directionality. The DV was the T value for each possible pair of hierarchies (i.e. prosody–syllable, prosody–phoneme and syllable–phoneme) and the within-language factor was directionality (top-down versus bottom-up). The Friedman repeated measures ANOVA for directionality indicated that the top-down information dynamics in the AM hierarchy were stronger than the bottom-up information dynamics in all of the pairs of hierarchies (the information dynamics from slower to faster speech rhythms, i.e. from prosody to syllable, from syllable to phoneme and from prosody to phoneme, *χ*² = 2300, *p *< 0.001). We then used non-parametric Kruskal–Wallis ANOVAs to compare values for German versus French. The DV was the T-value for each possible pair and directionality of hierarchies (i.e. prosody → yllable, syllable → prosody, prosody → phoneme, phoneme → prosody, syllable → phoneme and phoneme → syllable) and the between-group factor was language (German versus French). The Kruskal–Wallis ANOVA indicated that the T-value from syllable to phoneme, T (syllable → phoneme), was higher in French than in German (χ² = 11.16, *p* = 0.006, ε² = 0.02), again with a small effect size. Otherwise the T-values did not differ by language. The importance of top-down acoustic relations in metrical poems is depicted in figure 6, which shows the T statistics in each case.

## 4. Discussion

Metrical poetry in French and German is considered by linguists to be fundamentally different in rhythmic structure. The regular alternation of stressed and unstressed syllables, which is so familiar to those raised on German as well as English poetry, is not considered a significant part of the French poetic tradition. However, while metrical French poetry does not appear on the surface to foster a *salient* ‘beat’, we reasoned here that the phrasal accents used in French poetic forms may in fact create physical acoustic structures in the AE that are highly similar to the trochaic or *iambic* forms common in German poetry. To test our *a priori* hypothesis that the core physical stimulus characteristics, which define rhythm patterns in French versus German metrical poetry will be more similar than different, we applied a computational model of speech rhythm based on the speech envelope and the neural mechanisms of speech processing, the S-AMPH model [28]. We predicted that the number of bands of AM nested in the envelope and their boundaries would be very similar for French and German poems, and would match the AM bands identified previously for conversational English, Spanish and Portuguese [25,29,33]. We also predicted that there would be a similar set of phase relations (rhythmic synchronicity between AM bands) for the German and French poems, with the delta-rate and theta-rate AM bands exhibiting the largest PSIs in both languages.

In order to further characterize computationally the differences between French and German metrical poems that human listeners can hear, we also adapted computational metrics drawn from the comparison of rhythm patterns in human music versus birdsong [31,36]. This computational approach is not based on neural processing like the S-AMPH, but on musical analyses. It contrasts ‘horizontal rate’ patterns such as 1:1 (metronome-like) beat structures with ‘vertical ratios’, which identify the relations between cycles of rhythmic patterns at different hierarchical levels of a piece of music or the song of a bird. Our expectation was that French and German poetry might differ in terms of either the horizontal rate patterns, or the vertical ratio patterns, or both. We also adopted aspects of the neuroscience literature on transfer entropy in an attempt to analyse whether the metrical structure of one cycle of the prosody waveform could predict the metrical structure of the syllabic waveform that lies within the next cycle of the prosodic waveform. Given the use of syllable counting in French poetry and the use of lexical stress in German to create rhythm, we tentatively predicted that we would find higher top-down transfer entropy in German metrical poetry than in French metrical poetry.

Regarding the S-AMPH modelling, our findings indeed demonstrated the presence of similar hierarchical temporal structures in the AEs of French and German metrical poetry. As demonstrated in figures 2 and 3 and in table 3, the number of AM bands identified in the spoken forms and their boundaries were highly similar across languages, and the phase synchronization indices between the different bands were also extremely similar. The frequency bands identified in table 3 present an AM-driven perspective on the structural-acoustic elements of poetic rhythm. In particular, the band 1 AM frequencies (0.9–3 Hz), corresponding to word- or foot-level temporal patterns, support the integral role of metre in the foundation of poetic form. Our study used 0.9 Hz as the lowest frequency boundary based on the human cochlear filterbank [42,43]. Although frequencies below 0.9 Hz are present in rhythmic speech regardless of cochlear function, in a previous study [20] comparing cochlear and engineering-oriented models (S-AMPH versus the probabilistic amplitude demodulation model (PAD) [50]) we found that the most prominent frequency band for the slowest rhythms in speech was indeed between around 0.9 and 2 Hz (see fig. 2*d* in [20]). Nevertheless, the indication of potential peaks below 1 Hz in the PC3 results of figure 3 offers one avenue for further investigation. Our bandings depend in part on the neural literature, and there are also other prosodic patterns in poetry at longer timescales, such as the couplet. Accordingly, future modelling incorporating slower rhythmic elements below 1 Hz may also contribute to defining poetic metre. Future modelling that includes a wider range of genres and asks speakers to recite poems from memory as well as read the poems may help to expand the results.

Meanwhile, the present study extends the existing body of research on the AM characteristics of poetry by focusing on the presence of a beat in French poetry, indicated by the large and matching 1:2 delta-rate and theta-rate AM PSI shown for both French and German in figure 4. Prior modelling studies have suggested that this PSI could be a language-universal acoustic indicator of both speech and non-speech rhythm and ‘beat’ [20], thereby providing a ‘phase code’ for metrical rhythm [51]. The finding that French also showed the largest PSI between delta-rate and theta-rate AMs would not be expected if French metrical poetry were fundamentally different in linguistic structure from German metrical poetry. This discovery further aligns with the neural importance of temporal prediction in language processing, supporting a universal beat-based underpinning across different languages [52].

The horizontal rate and vertical ratio modelling revealed both similarities and differences between metric poetic structures in the two languages. Both languages showed essentially identical horizontal rate structure, as can be seen in electronic supplementary material, appendix S5. Again, this indicates highly similar prosodic structures. Both languages exhibited a 1:1 horizontal rate within the same acoustic hierarchy, which could be argued to represent the beat. However, the probability densities of the 1:2 vertical ratio were significantly stronger in German than in French poetry, albeit with a small effect size. This indicates that German metrical poetry is slightly more likely to contain syllabic cycles that are half of a prosodic cycle compared to French poetry. Temporally, this would have the effect of strengthening the perceived regularity of the ‘beat’ for German. Another difference, again with a small effect size, was revealed by the transfer entropy analyses. French poetry showed a greater top-down relationship between syllables and phonemes than German poetry. This may imply that the number of phonemes that can comprise syllables is more constrained in French metrical poems. Nevertheless, the transfer entropy results suggested that the ‘flow’ of information within the hierarchical structure of metrical poems is top-down controlled, regardless of language. This implies that prosody exerts control over the lower-level rhythmic structure of syllable placement in both languages.

The similarities and differences revealed by our modelling align with previous research suggesting that human auditory signals, including adult speech, song and music, contain universal AM and rhythmic rate patterns [20,25,28,29,31,33,36]. These universals comprise similar temporal structure in terms of AM bandings, which were identical for German and French, and AM phase relations, which were also essentially identical for German and French. Accordingly, the acoustic structures created by the more mobile accents in French poetry bear high physical similarity to trochaic or iambic forms at the level of the acoustic signal. This suggests core neural similarities in the perception and production of oral rhythmic structures across languages. However, two differences are of note. The 1:2 vertical ratio between different acoustic bandings was stronger in German, indicating that the length of a syllabic cycle is more likely to be half of a prosodic cycle compared to French, and the top-down transfer entropy from syllables to phonemes was significantly stronger in French, indicating greater syllabic control over the phonemes comprising the syllables.

These findings allow us to add some nuance to the linguistic analyses of French poetic rhythms quoted at the start of this article.

‘The French accent falls on the last accentuable syllable of each syntactic unit in the line, and since these units naturally vary in length, French rhythmic measures obey no law of recurrence and no principle of regularity, and thus have no connection with the notion of beat’ [3, p. 198].

Our language-blind computational analyses show that there is both recurrence and regularity in French rhythmic measures. The law of recurrence is stronger in the German corpus, as indicated by the larger 1:2 vertical ratio, but the principle of regularity is the same in both sets of poems, as shown by the delta–theta PSI and the 1:1 horizontal rates. Furthermore, the end-of-phrase accents in French noted by Scott [3] do not necessarily have to be irregular. Natural speech often incorporates complex ‘polyrhythms’ to achieve more uniform phrase-level accents [53]. Such structures aim to align quasi-periodic delta-level periodicities with syntactic phrase termini, thereby enhancing cognitive processing [54]. Hilton and Goldwater argue that this rhythmic phenomenon likely extends to many languages, representing a universal aspect of linguistic rhythm influenced by syntax-aligned phrase structures. Our analyses show that the predictability of recurring intervals is equally strong in both German and French poems, as the top-down driven (transfer entropy) statistics are same. However, French differs from German in that it may exert timing control through the number of phonemes that comprise syllables. Our dataset therefore supports the view that there are differences between the poetic structures of the two languages, but also reveals many clear similarities.

The most significant finding from our modelling is that the delta–theta PSI is the most prominent in both languages. The 1:2 integer ratio found here is entirely predictable for the German corpus which, as emphasized in §2.2 of this article, is composed of bi-syllable prosodic feet, which produce a regular alternation between stressed and unstressed syllables. The finding that this 1:2 ratio is also present in the French corpus, where the number of syllables between accents is much more variable, is both novel and important. This finding is not unprecedented poetically, in that a prior analysis of English poetry written for children (English nursery rhymes) included metrical structures with both bi-syllable and tri-syllable feet, and also yielded a 1:2 integer ratio [28]. Yet, it is particularly striking in relation to classical French poetry, because it indicates that there is after all a beat structure in French poetry. The phase alignment of the delta- and theta-rate AM bands in French poems would not be expected on some linguistic analyses to be identical to German. The finding that the 1:2 integer ratio was dominant in the delta–theta PSI in both languages suggests that, biophysically, this enables the perceptual extraction of the beat. It is worth noting that the 1:2 delta–theta PSI has also characterized previous studies of beat placement across various rhythmic genres, including music, song and IDS and CDS in various languages [20,25,29]. This PSI is not so clearly observed in conversational speech [29], particularly for ADS, and particularly for illiterate participants [33]. Finally, the fact that the 1:2 *vertical* ratio is stronger in German shows that the timing of the beat placement is more strictly controlled in that language, which, again, we would expect from prior linguistic analyses; but on balance, the similarities between the two languages, as shown by the delta–theta PSI and the horizontal ratio, are more evident than the differences.

Although the presence of a beat in French poetry challenges our received understanding of how versification works, it resonates strongly with a psychological given, namely the neural importance of temporal prediction in language processing [52]. Temporal prediction is crucial for speech processing, because the brain uses temporal features of the incoming signal to predict when the next important piece of information will occur. In metrical poetry, that predictability is heightened into the regular beat structure which is particularly salient in German (and indeed English), but, as we have now demonstrated, is also present in French. The returning accent of French poetry thus has a fundamentally similar physical function to the stressed syllables of German verse.

There are some limitations to the present work. While our AM- and periodicity-based analyses highlight key acoustic similarities in French and German metrical poetry, they may not capture the entirety of rhythmic characteristics inherent in these linguistic traditions. Our modelling approach may inadvertently obscure qualitative differences that are equally vital perceptually but which manifest at different levels of poetic structure or aesthetic appreciation. Other approaches, such as linguistic patterning, semantic weighting or cultural–historical contexts, may reveal such differences. A further question that arises from our findings is how to describe the perceptual effects of the dominant 1:2 ratio for the delta–theta AM PSI. Our prior approach for CDS was to assume that the parent element typically encompasses two daughter elements for the rhythm—so, for example, two syllables per prosodic foot. This works for the duple rhythms in our German corpus, but it cannot apply to the French poems: the number of syllables (i.e. daughter elements) per ‘measure’ (parent element) is highly variable. Moreover, we have already seen that an English corpus which includes triple rhythms (i.e. three syllables or daughter elements per prosodic foot) also yields a 1:2 ratio [28]. Thus our analysis also suggests that we need to conceive of the daughter elements differently when analysing the complex linguistic structures of metrical poetry. Here, the strong beat indicated by the 1:2 ratio cannot be simply matched to linguistic units such as individual syllables. Another limitation is that there are likely to be acoustic differences between the readings by the lay participants in our study and acoustic approaches that may be taken by poets or other poetry professionals (teachers and actors). Furthermore, reading poetry aloud from the text differs from reciting poetry from memory. Therefore, our findings may be specific to the genres and speaker types examined.

In conclusion, our computational modelling of recited French and German metrical poems contributes to our understanding of the fundamental temporal structures underlying rhythmic organization in speech. The modelling is suggestive of the presence of a shared hierarchical AM structure in metrical poems in the two languages, with matching AM phase relations providing the acoustic statistics underlying rhythm structures in both genres. However, the modelling also highlighted the role of the 1:2 vertical ratio in shaping rhythmic organization in German, yielding a more tightly controlled ‘beat’, and the syllable-to-phoneme transfer entropy in shaping rhythmic organization in French, where it potentially aids temporal processing by restricting the number of phonemes that can comprise syllables. Poetry, as a form of musical language, appears to tap into the temporal structures that facilitate the neural processing of both rhythmic speech and music, even though the linguistic realization of these structures may appear to vary across languages.

## Data Availability

All of the anonymized raw data files have been deposited to an external source [55]. The other data and results of statistical analysis are available in electronic supplementary material [56].
